# Recurrent Hypertensive Carcinoid Crisis Despite Somatostatin Analog Therapy

**DOI:** 10.7759/cureus.119532

**Published:** 2026-11-12

**Authors:** Ameen Ghannoum, Amy-Claire Young, Ranganatha Rao

**Affiliations:** 1 Diabetes and Endocrinology, University Hospitals Coventry and Warwickshire NHS Trust, Coventry, GBR

**Keywords:** carcinoid crisis, carcinoid syndrome, ga-68 dotatoc pet/ct, hypertensive carcinoid crisis, lanreotide, neuroendocrine tumor, octreotide, somatostatin analogs

## Abstract

Carcinoid crisis is a rare, life-threatening complication of neuroendocrine tumors characterized by hemodynamic instability. Although hypotension is more commonly described, severe hypertension can also occur. An 84-year-old woman who had undergone resection of a grade 1 terminal ileal neuroendocrine tumor and two hepatic metastases presented with worsening flushing, severe headaches, mild wheeze, palpitations, and chest tightness. She was receiving maintenance long-acting lanreotide and had used the maximum prescribed dose of rescue subcutaneous octreotide for two weeks without sustained benefit. Her blood pressure was highly labile, with a peak systolic reading of 198 mmHg, and her heart rate reached 110 beats/min. The endocrinology team diagnosed a hypertensive carcinoid crisis. Her symptoms and hypertension resolved within 24 h of commencing a continuous intravenous octreotide infusion, and a plan was made to shorten the lanreotide dosing interval from three weeks to two weeks. Eleven days after discharge, she was readmitted with recurrent flushing, palpitations, tachycardia, and hypertension after the dose due under the revised lanreotide schedule was delayed. Subcutaneous octreotide improved the flushing and hypertension, while bisoprolol was introduced for persistent symptomatic tachycardia. Cardiac investigations showed no sustained arrhythmia or major structural abnormality. Biochemical markers measured after treatment were within their reference ranges. Subsequent somatostatin receptor positron emission tomography/computed tomography demonstrated new receptor-avid disease in the liver and left pelvis that had not been identified on preceding conventional imaging. This case demonstrates that hypertension can be the predominant hemodynamic manifestation of carcinoid crisis and highlights the limited value of normal biomarkers obtained after somatostatin analog treatment, which should not be used in isolation to exclude an acute crisis.

## Introduction

Carcinoid syndrome results from the systemic effects of serotonin and other vasoactive mediators released by functioning neuroendocrine tumors. Classic features include cutaneous flushing and diarrhea, although bronchospasm, hemodynamic instability, and carcinoid heart disease may also occur [1]. Maintenance treatment relies on long-acting somatostatin analogs, such as lanreotide, while short-acting octreotide may be used for breakthrough symptoms [1]. Lanreotide and octreotide bind to somatostatin receptors on neuroendocrine tumor cells and inhibit the secretion of hormones and vasoactive substances, thereby reducing hormone-mediated symptoms [1].

Carcinoid crisis is an uncommon, potentially life-threatening deterioration marked by abrupt hemodynamic instability, often accompanied by flushing, bronchospasm, or arrhythmia [1,2]. Its pathophysiology remains incompletely understood but may reflect the sudden systemic effects of tumor-derived vasoactive mediators, whose variable effects on vascular tone can produce hypotension or hypertension [2,3]. It is usually described as a hypotensive event, particularly around surgery, although severe hypertension is recognized [1-3]. Spontaneous hypertensive carcinoid crisis outside procedural settings remains sparsely reported, with published evidence largely limited to individual case reports [2,3]. There is no universally accepted diagnostic definition [2,4], and biochemical markers cannot reliably confirm or exclude an acute episode [1,2]. We present a case of recurrent hypertensive carcinoid crisis occurring despite maintenance lanreotide and frequent rescue octreotide, highlighting the diagnostic challenge posed by an atypical hemodynamic presentation and the need for timely treatment escalation.

## Case presentation

An 84-year-old woman had initially presented approximately nine years earlier with mechanical small-bowel obstruction. Diagnostic assessment identified a well-differentiated grade 1 neuroendocrine tumor of the terminal ileum and two small hepatic metastases. She underwent laparotomy, right hemicolectomy, and resection of both liver lesions with perioperative octreotide cover. Histopathology confirmed a well-differentiated grade 1 ileal neuroendocrine tumor with a Ki-67 index below 1%. The primary ileal tumor was staged as pT4 N1, with an R1 margin and involvement of five of 10 regional lymph nodes. Both resected liver lesions contained metastatic well-differentiated neuroendocrine tumor. She remained on long-acting lanreotide after surgery. Approximately two years before the current presentation, because of breakthrough symptoms, her maintenance regimen was adjusted to lanreotide 120 mg every three weeks alongside rescue subcutaneous octreotide.

Surveillance magnetic resonance imaging of the liver and subsequent computed tomography of the thorax, abdomen, and pelvis performed before the acute presentations showed no evidence of recurrent hepatic disease or other disease progression on conventional imaging. Her medical history included essential hypertension, which was usually controlled with amlodipine 5 mg and ramipril 10 mg once daily.

First admission

During the first admission, she presented with a one-week history of worsening flushing and tachycardia. During the preceding two weeks, she had required the maximum prescribed rescue dose of subcutaneous octreotide, 100 μg three times daily, without sustained benefit. She subsequently developed severe headaches that woke her from sleep, mild wheeze, and one episode of vomiting. On the morning of admission, she also experienced palpitations and mild chest tightness. Paramedics found her severely hypertensive and brought her to hospital.

Her systolic blood pressure on admission was 198 mmHg and remained labile, ranging from 111 to 198 mmHg, although readings were frequently around 170 mmHg. Her heart rate was generally 80-110 beats/min, compared with a usual resting rate in the 70s. Cutaneous flushing and mild wheeze were confirmed on examination; the remainder of the relevant examination was unremarkable. Hypertensive emergency is defined as severe hypertension accompanied by acute hypertension-mediated target-organ damage, rather than by the blood-pressure value alone [5]. Although her systolic blood pressure reached 198 mmHg, no acute hypertension-mediated target-organ damage was identified; therefore, the episode was consistent with hypertensive urgency rather than hypertensive emergency. In view of the progressive carcinoid symptoms, acute hemodynamic disturbance, and failure of maximum rescue octreotide, the endocrinology team diagnosed a hypertensive carcinoid crisis.

A continuous intravenous octreotide infusion was administered at 12.5 μg/h for 24 h. Her flushing, headaches, palpitations, and hypertension resolved within that period without adjustment of her usual antihypertensive medication. Following specialist review, the lanreotide dosing interval was shortened from three weeks to two weeks because symptom control was no longer sustained throughout the three-week interval despite rescue subcutaneous octreotide, and she was discharged.

Biochemical testing performed after octreotide treatment showed 5-hydroxyindoleacetic acid and chromogranin A within their respective laboratory reference ranges. As the samples were collected after intensive exposure to short- and long-acting somatostatin analogs, the normal results were not considered sufficient to exclude the diagnosis. Results of 24-h ambulatory electrocardiographic monitoring showed sinus rhythm with intermittent first-degree and Mobitz type I atrioventricular block and occasional multifocal ventricular ectopy, but no sustained arrhythmia to explain her symptoms. Thyroid function tests and metanephrines were within their respective laboratory reference ranges during both admissions, making thyrotoxicosis and pheochromocytoma less likely.

Second admission

After discharge, she continued subcutaneous octreotide 100 μg three times daily. At 11 days postdischarge, she was readmitted with recurrent flushing and palpitations after the dose due under the revised lanreotide schedule was delayed because the medication was unavailable. Lanreotide was not administered during this admission, and further intravenous octreotide infusion was not given. Her systolic blood pressure was 173 mmHg on presentation and reached 177 mmHg, remaining mostly above 140 mmHg during the earlier part of the admission. Her heart rate was 107 beats/min on arrival, with further episodes of symptomatic tachycardia.

The responsible consultant diagnosed a recurrent carcinoid crisis. Subcutaneous octreotide 100 μg three times daily was continued, with improvement in flushing and hypertension. Bisoprolol was introduced separately for persistent symptomatic sinus tachycardia and palpitations and was titrated to 5 mg once daily. Cardiac troponin was normal. Transthoracic echocardiography showed a left ventricular ejection fraction of 60-65%, without major structural or valvular abnormality. Her blood pressure and heart rate returned to their usual baseline ranges, with no further palpitations during the final three days before discharge. The delayed lanreotide dose was administered following discharge.

No further crises or hospital admissions had been documented at the latest follow-up. Compared with the previous somatostatin receptor positron emission tomography/computed tomography, subsequent gallium-68 DOTA-D-Phe1-Tyr3-octreotide (DOTATOC) positron emission tomography/computed tomography demonstrated longstanding focal uptake in the sigmoid colon, a new left obturator lymph node or peritoneal nodule, new focal uptake in the right hepatic lobe, and a new small avid right middle lobe pulmonary nodule that was considered suspicious (Figures 1A-1D). The new abnormalities had not been identified on the preceding conventional imaging.

**Figure 1:**
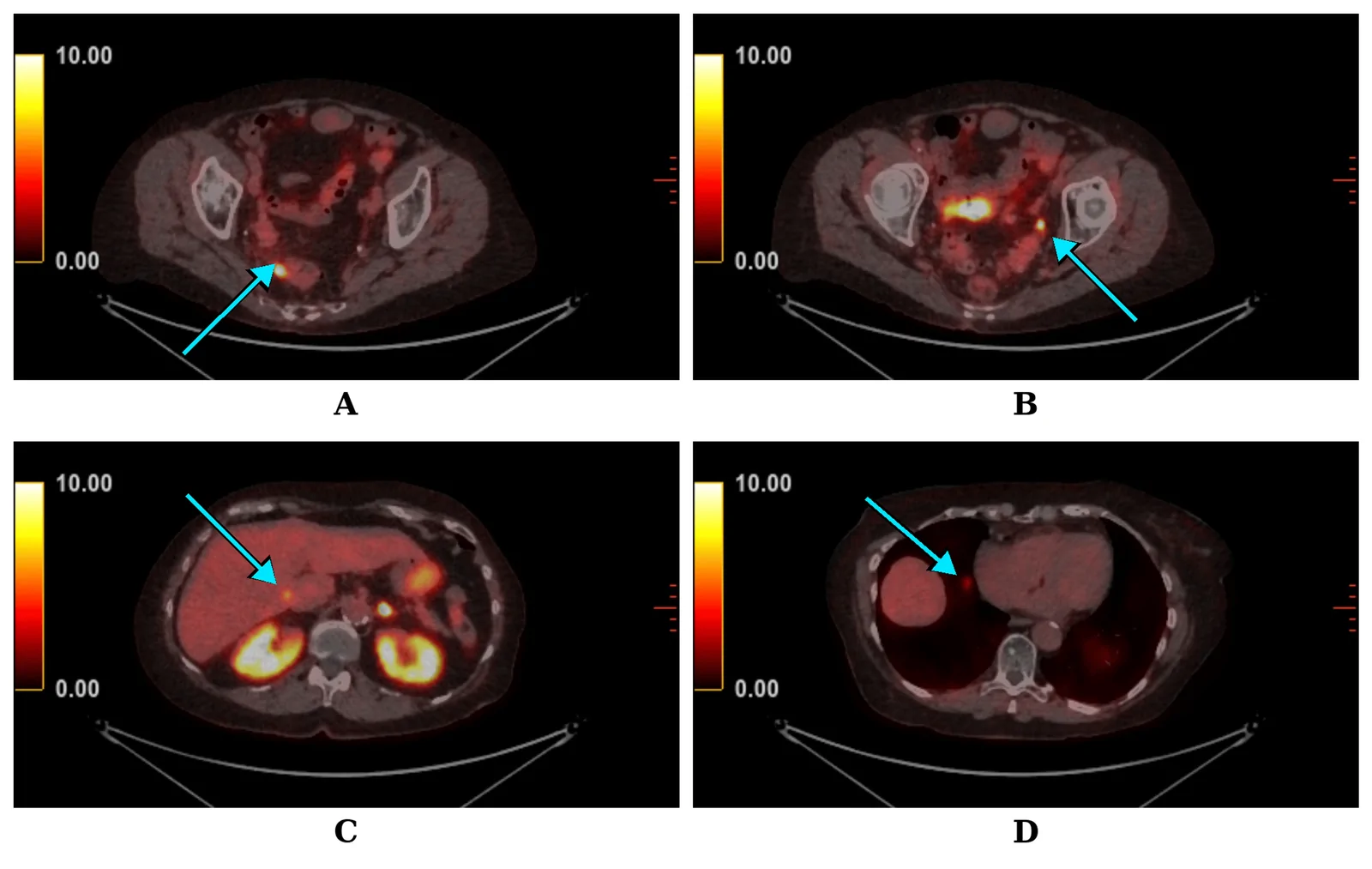
Gallium-68 DOTATOC PET/CT findings. Fused axial PET/CT images acquired using an Omni Legend 32 PET/CT scanner (Waukesha, WI: GE HealthCare). Cyan arrows indicate (A) focal tracer uptake in the sigmoid colon (SUVmax: 26.11); (B) focal tracer uptake along the left pelvic sidewall, corresponding to a left obturator lymph node or peritoneal nodule (SUVmax: 19.33); (C) focal tracer uptake in the right hepatic lobe (SUVmax: 4.40); and (D) focal tracer uptake in the right middle lobe of the lung (SUVmax: 4.22). Images were acquired 58 min after intravenous administration of 134 MBq of gallium-68 DOTATOC and reconstructed using Q.Clear (Waukesha, WI: GE Healthcare). SUVmax: maximum standardized uptake value; DOTATOC: DOTA-D-Phe1-Tyr3-octreotide

## Discussion

Distinguishing carcinoid crisis from an exacerbation of essential hypertension can be challenging. In this patient, hypertension had previously been controlled on stable treatment. The temporal association of labile hypertension with flushing, wheeze, and palpitations, together with their rapid resolution following intravenous octreotide without adjustment of her usual antihypertensive medication, supported the diagnosis of hypertensive carcinoid crisis [3].

There is no universally accepted definition of carcinoid crisis, and most quantitative evidence is derived from perioperative studies [2,4]. Condron et al. examined intraoperative carcinoid crises, whereas Fouché et al. classified broader episodes of intraoperative carcinoid syndrome [6,7]. Their differing definitions and hemodynamic patterns are summarized in Table 1 [6,7]. These studies do not provide incidence estimates for spontaneous crisis, but they demonstrate how differences in case definition can influence whether hypotensive or hypertensive intraoperative events predominate.

**Table 1:** Comparison of perioperative studies illustrating heterogeneity in definitions of intraoperative carcinoid events.

Study and setting	Definition or classification	Principal findings	Relevance to this case
Condron et al. [6] - prospective case series: 127 patients undergoing 150 operations with continuous octreotide infusion	Intraoperative crisis was characterized predominantly by sustained hemodynamic instability and hypotension.	Crises occurred in 30% of operations despite octreotide infusion. Prompt vasopressor treatment shortened the duration of hypotension.	Demonstrates the predominance of hypotension when a narrower perioperative definition is applied and that prophylactic octreotide does not eliminate intraoperative events.
Fouché et al. [7] - retrospective series: 81 patients undergoing small-bowel neuroendocrine tumor surgery with perioperative octreotide	A highly probable intraoperative carcinoid syndrome episode required rapid hemodynamic change of at least 40%, unexplained by surgical or anesthetic management, with at least 20% regression after octreotide. Probable and suspected episodes used broader criteria.	There were 139 intraoperative carcinoid syndrome episodes in 45 patients. Episodes were hypertensive in 91% and/or hypotensive in 29%, with overlap between categories. No episode met the authors' definition of intraoperative carcinoid crisis.	Demonstrates that broader definitions of intraoperative carcinoid events can identify predominantly hypertensive episodes; however, these events were not classified as carcinoid crises by the authors, and the study cannot establish their frequency in spontaneous crisis.

The predominance of perioperative evidence also limits its application to crises occurring outside procedural settings. Carcinoid crisis is most frequently reported following anesthesia, surgery, tumor manipulation, embolization, or peptide receptor radionuclide therapy, although spontaneous episodes are recognized [2]. Published evidence specifically addressing spontaneous hypertensive carcinoid crisis remains sparse and is largely limited to individual case reports [2,3]. No clear precipitating trigger was identified during this patient’s first admission. The second episode followed a delay in her scheduled lanreotide dose, which may have contributed to reduced symptom control and recurrence, although the temporal association does not establish causation. The pathophysiology of carcinoid crisis remains incompletely understood but is thought to involve the sudden systemic effects of tumor-derived vasoactive mediators [2]. Their variable effects on vascular tone may account for presentations ranging from profound hypotension to severe hypertension [2,3].

The normal 5-hydroxyindoleacetic acid and chromogranin A results must be interpreted in relation to their timing, as both were measured after treatment with short- and long-acting somatostatin analogs. These biomarkers reflect selected secretory products and are not real-time measures of every vasoactive mediator potentially involved in a crisis [1,2]. The results therefore neither confirmed nor excluded the acute diagnosis, and normal post-treatment biomarkers should not be used in isolation to exclude carcinoid crisis when the clinical features are supportive.

Intravenous octreotide is central to the acute management of carcinoid crisis, alongside supportive cardiovascular treatment [1,2]. Current UK and Ireland Neuroendocrine Tumour Society (UKINETS) guidance is summarized in Table 2 [8]. During the first admission, the patient's symptoms and hypertension resolved within 24 h of commencing intravenous octreotide. The infusion rate used in this case was lower than the regimen described in the current UKINETS guidance but was followed by rapid clinical resolution. During the second admission, no further infusion was given. Clinical improvement occurred while subcutaneous octreotide was continued and bisoprolol was introduced for persistent symptomatic sinus tachycardia and palpitations.

**Table 2:** Summary of UKINETS guidance for the acute management of carcinoid crisis. UKINETS: UK and Ireland Neuroendocrine Tumour Society

Clinical component	Recommended management
Hemodynamic instability	Request early support from the critical care team.
Somatostatin analog	Administer intravenous octreotide 100 μg as a bolus, repeated if required, followed by an infusion at 50 μg/h.
Monitoring	Monitor for hyperglycemia and bradyarrhythmias during the octreotide infusion.
Additional medication	Administer intravenous hydrocortisone 100 mg and intravenous chlorpheniramine 10 mg.
Hypotension	Give intravenous fluids and consider phenylephrine, noradrenaline, or vasopressin.
Hypertension	Optimize analgesia and consider intravenous magnesium or glyceryl trinitrate infusion in the acute setting.
Medication precautions	Avoid agents associated with histamine or serotonin release.

Before the first admission, the patient had experienced increasing breakthrough symptoms despite using the maximum prescribed dose of rescue octreotide. This resembles the clinical pattern described in refractory carcinoid syndrome, in which symptom control may diminish two to three weeks after administration of a long-acting somatostatin analog and deteriorate before the next injection [9]. In this patient, the possibility of inadequately controlled or refractory carcinoid syndrome remained, although biochemical interpretation was limited because 5-hydroxyindoleacetic acid and chromogranin A were measured after intensive somatostatin analog treatment. The normal post-treatment results did not exclude inadequately controlled hormone-mediated symptoms. Increasing the dose or frequency of a somatostatin analog, or switching within the drug class, has been associated with improvement in flushing and diarrhea in 72-84% of reported cases [9,10]. These findings support early specialist reassessment when breakthrough symptoms become more frequent and provide context for shortening her lanreotide interval from three weeks to two weeks.

The two hepatic metastases present at the original diagnosis had been resected, and subsequent surveillance magnetic resonance imaging and computed tomography showed no evidence of recurrent hepatic disease or other progression on conventional imaging. Compared with the previous somatostatin receptor positron emission tomography/computed tomography, the later gallium-68 DOTA-D-Phe1-Tyr3-octreotide (DOTATOC) positron emission tomography/computed tomography demonstrated new somatostatin receptor-expressing disease in the right hepatic lobe and a left obturator lymph node or peritoneal nodule. These findings represented new receptor-avid abnormalities rather than persistence of the original resected liver lesions. They supported the presence of an active neuroendocrine tumor but did not, in isolation, establish that the acute episodes represented carcinoid crisis.

## Conclusions

This case illustrates that hypertension may be the predominant hemodynamic feature of a carcinoid crisis, including outside perioperative settings. Clinicians should maintain a high index of suspicion when patients with neuroendocrine tumors present with labile hypertension accompanied by classic vasoactive symptoms, even in the setting of previously controlled essential hypertension. In the absence of universally accepted diagnostic criteria, the diagnosis remains clinical and requires consideration of alternative causes. Normal biochemical markers obtained after somatostatin analog treatment should not be used in isolation to exclude a crisis. Increasing breakthrough symptoms or rescue octreotide requirements should prompt early specialist reassessment and optimization of maintenance therapy. Prompt specialist review and escalation of somatostatin analog treatment were followed by clinical improvement in this patient. The temporal association between recurrence and the delayed scheduled lanreotide dose did not establish causation but nevertheless highlights the importance of reliable access to long-acting maintenance treatment following discharge.
